# Integrated platform and API for electrophysiological data

**DOI:** 10.3389/fninf.2014.00032

**Published:** 2014-04-23

**Authors:** Andrey Sobolev, Adrian Stoewer, Aljoscha Leonhardt, Philipp L. Rautenberg, Christian J. Kellner, Christian Garbers, Thomas Wachtler

**Affiliations:** Department Biology II, German Neuroinformatics Node, Ludwig-Maximilians-Universität MünchenPlanegg, Germany

**Keywords:** electrophysiology, data management, neuroinformatics, web service, collaboration, neo, odml

## Abstract

Recent advancements in technology and methodology have led to growing amounts of increasingly complex neuroscience data recorded from various species, modalities, and levels of study. The rapid data growth has made efficient data access and flexible, machine-readable data annotation a crucial requisite for neuroscientists. Clear and consistent annotation and organization of data is not only an important ingredient for reproducibility of results and re-use of data, but also essential for collaborative research and data sharing. In particular, efficient data management and interoperability requires a unified approach that integrates data and metadata and provides a common way of accessing this information. In this paper we describe GNData, a data management platform for neurophysiological data. GNData provides a storage system based on a data representation that is suitable to organize data and metadata from any electrophysiological experiment, with a functionality exposed via a common application programming interface (API). Data representation and API structure are compatible with existing approaches for data and metadata representation in neurophysiology. The API implementation is based on the Representational State Transfer (REST) pattern, which enables data access integration in software applications and facilitates the development of tools that communicate with the service. Client libraries that interact with the API provide direct data access from computing environments like Matlab or Python, enabling integration of data management into the scientist's experimental or analysis routines.

## 1. Introduction

### 1.1. Data management in electrophysiology—costs, benefits, and needs

Advances in technology and methodology during the past years have dramatically increased the volume and complexity of data recorded in electrophysiological experiments. At the same time, progress in neuroscience increasingly depends on collaborative efforts, exchange of data, and re-analysis of previously recorded data. Thus, ensuring that data stays accessible, that data processing is reproducible, and that data can be shared and re-used has become a challenge for many laboratories (Herz et al., 2008).

**Figure 1:**
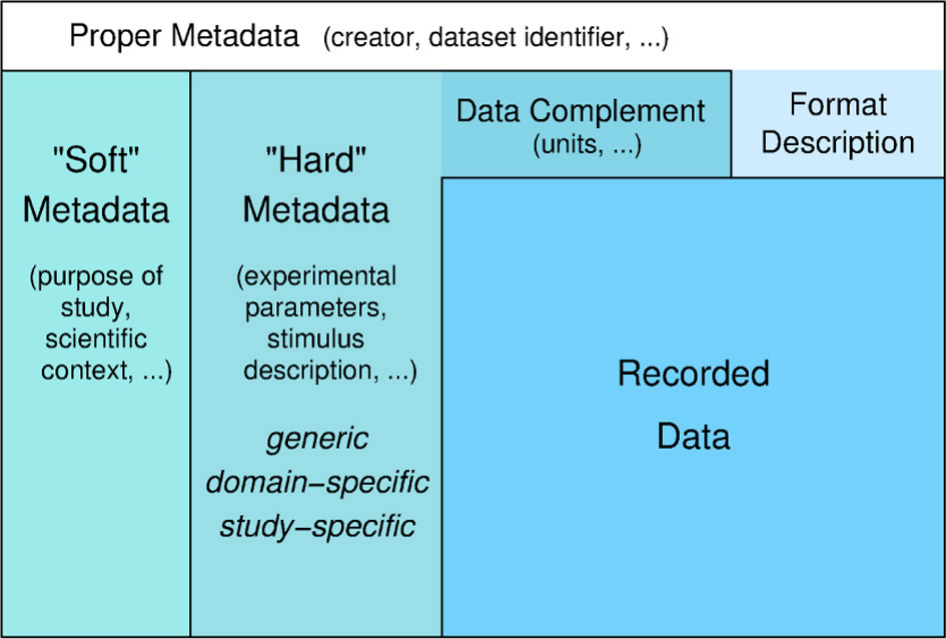
**Levels of (meta)data**. Recorded data and additional information that is necessary for understanding and appropriate analysis of the data. Information about the format in which the data are stored is required to read the data. Information that complements the raw stored numbers, such as sampling rate, scaling factors, units, is required to understand the data as measured signals. To meaningfully analyze the data, information about the experimental context is necessary, like conditions of preparation, stimulation, etc. This information in principle can be formalized and stored in machine-readable form (“hard metadata”) so that it can be used for data selection and analysis. This metadata can be further categorized into generic, domain-specific, and study-specific information. “Soft metadata” is the information about the overall scientific context and aim of the study, reasons for choosing certain parameters, etc., for which currently we have no way of formalizing or machine-processing. The distinction between supplementing data and proper metadata is to some degree arbitrary. For example, the date when an experiment was performed might usually be considered as proper metadata. However, in some analysis the time between experiments might be an important parameter to be taken into account. In this case, the date of the experiments can be used as data in the analysis to determine this information.

Obstacles to efficient data management arise not only from the variety of data formats and constraints of accessing data in proprietary formats, but also from the amount and complexity of additional information about the experiment that needs to be collected and stored. This additional information, which is commonly called “metadata” despite the fact that it is to large part data supplementing the recorded data (Figure 1), is not only necessary to reproduce the study but also essential for searching, selecting, and analyzing the data.

Collecting and storing metadata comprehensibly together with the recorded data is also a facilitating requisite for sharing the data. Data sharing starts in the lab, where data needs to stay accessible and understandable for the experimenter even years after the study, and lab members need to be able to find and access data even after the person that performed the experiment has left the lab. In collaborations with scientists outside the laboratory, data need to be selected and the collaborators need to be able to understand the data. Having a data organization in the lab where all data and metadata is kept together in defined formats and organized structure can reduce both the experimenter's work for data preparation and the collaborator's efforts to read and understand the data. In the same way, efficient data organization minimizes the time and work necessary for preparing data to make it generally accessible, thus reducing the barriers to public sharing and data publication.

Experimental metadata typically have to be collected from various sources and in different formats—different measurement devices, software code, notes entered during the experiment, etc.—and have to be brought into compatible formats, which can require considerable effort. Typically, each lab defines its own methods, procedures, and format conventions for organizing and managing the data. If common tools and formats were available, workload and time demand in the labs would be reduced and data exchange would require less effort and time.

Developing common tools and standardized formats has turned out to be particularly challenging for the area of electrophysiology (Teeters et al., 2013). This field faces an enormous variety in experimental methodology, with a large number of data acquisition systems, file formats that are often vendor-specific and undocumented (Garcia et al., 2014), a variety of electrode configurations, species, preparations, stimuli, and overall experimental paradigms. Currently, common organization schemes or standards for accessing data do not exist. Thus, for data exchange, often substantial work is necessary to make the data accessible in one form or another. Moreover, in electrophysiology the experimental variety and complexity results in corresponding variety and complexity of the metadata. While a set of minimal common metadata for a neuroscience experiment has been proposed (Gibson et al., 2008), for each dataset further specific information needs to be provided. As long as a comprehensive ontology for this field is missing (Bandrowski et al., 2013), approaches to achieve a common scheme for metadata description must leave sufficient flexibility to account for the variety and heterogeneity of experiments (Grewe et al., 2011).

### 1.2. Databases and sharing platforms for electrophysiological data

In the past years, several initiatives to support data sharing in neurophysiology have emerged. One of the first public databases for electrophysiological data was the neurodatabase.org^1^ project (Gardner, 2004). In this project, an elaborate data model and format along with a query protocol for the exchange of neurophysiological data were developed. The data, typically obtained from publications, is made available with extensive metadata and provided in a format specifically developed for this project.

The SenseLab Project^2^ is a long-term initiative to build a repository for multidisciplinary models of neurons and neural systems (Crasto et al., 2007). It is a part of the Neuroscience Information Framework^3^ (NIF, Gardner et al., 2008) and the International Neuroinformatics Coordinating Facility (INCF)^4^. The project provides open databases (ModelDB, NeuronDB, etc.) designed for certain aspects like neural modeling, neural cell properties, modeling of neurocircuits and several others.

The CRCNS.org site^5^ hosts electrophysiological data that have been specifically selected by contributing labs for the purpose of making the data available to the public (Teeters et al., 2008). Typically, these data are from published studies and have been made available for re-use. Data format, annotation and documentation are different for each dataset.

The CARMEN project^6^ provides a platform for data analysis and data exchange where the owner of the data can keep the data private, or can make the data available to selected users or the public. The platform also provides services for data analysis (Austin et al., 2011). For this purpose Carmen has introduced an internal file format, Carmen NDF^7^, that is suitable for storing electrophysiological and other types of neuroscientific data. The user has the option to enter metadata describing the experiment in which the data were recorded. This is done via web forms that provide fields corresponding to the minimal metadata that were proposed by the Carmen consortium (Gibson et al., 2008).

The German Neuroinformatics Node (G-Node) provides a platform for data organization and data sharing of neurophysiological data^8^. Users can upload, organize, and annotate their data, and make them accessible to selected users or the public. Data conversion functions are provided. Data annotation follows a flexible schema (Grewe et al., 2011) so that any metadata necessary can be entered.

Recently, the INCF established the INCF Dataspace^9^, a cloud based file system to federate all kinds of neuroscience data. There are several other initiatives (Marcus et al., 2007; Usui and Okumura, 2008; Moucek et al., 2014, etc.) that provide a web-based storage for different domains in neuroscience.

All these solutions are based on data exchange by files, and they provide little or no support for using formats or data structures that are in some way standardized. In most cases data are accessible only through a web browser. Interoperability between any of these solutions, or with other tools and formats used by neuroscientists, does not exist. As a basis for such interoperability, common standards for representing and accessing data would be needed, and tools and services to apply and use these standards also within the lab would have to be available. Such standardization will become also highly relevant for the recently initiated large-scale projects with strong electrophysiology components, such as the Allen Institute's Project Mindscope^10^, the Human Brain Project^11^, or the BRAIN Initiative^12^.

Here we present GNData, the new version of the G-Node data management platform. This advanced version was developed with the aim not only to set up a repository of data files, but to provide a comprehensive framework that scientists can use to manage, access, and work with their data within their local laboratory workflow. The principal novelty of this framework is a standardized Application Programming Interface (API) together with client tools that enable data access directly from the local computational and/or laboratory environment. A unique feature is the ability to store and organize both the recorded data and the metadata together so that all information necessary for data analysis, re-analysis, and sharing is available in a unified way, and accessible through a well-defined interface. The integration of data and metadata has the benefits that data handling in the laboratory from recording to analysis becomes more efficient and reproducible, and that data sharing requires no further effort because all the information is already available with the data. Additionally, GNData allows for data sharing with colleagues, collaborators, or the public without any obstacles.

## 2. Approach

GNData addresses the need for comprehensive data management by providing (1) a storage system based on a common data representation that is suitable to organize data and metadata from any electrophysiological experiment, with (2) a general API so that data access can be integrated in software applications, and (3) client tools in common languages to support and facilitate this integration into the laboratory data workflow. These components implement a unique and efficient way of experimental data and metadata management, compared to the file-based systems.

### 2.1. Representation of data objects

A key element supporting reproducibility and data sharing is the standardization of formats and data structures. Using common data objects facilitates data access and data exchange, as well as the application of analysis tools. However, to be useful, standards must be applicable to the entire field without constraining the ability to store what is necessary. Given the variety and heterogeneity of electrophysiological studies, this poses the challenge of finding a balance between strict definitions to achieve the necessary standardization and flexible methods to account for the needs of any use case. GNData achieves this balance by combining a fixed data model for the recorded data with an adaptable and maximally unconstrained format for the metadata.

For the representation of electrophysiological data, the Neo python objects^13^ are widely used (Garcia et al., 2014) and have come close to being a de-facto standard for describing recorded electrophysiological signals. Neo defines an object model with attributes and relationships that accounts for all types of recorded data (signal and spike data, multi-electrode data etc.), including numerical values, units, and dimensions. A typical Neo experimental representation is a dataset (named *Block* in Neo) containing several experimental trials (*Segments*), each having time series (*AnalogSignals*), spike event data (*SpikeTrains*) and stimulus event times as Neo *Events*. A dataset (*Block*) usually also contains groups of electrodes (*Recording Channel Groups, Recording Channels*) related with the recorded signals to indicate spatial position and arrangement of electrodes, and units (*Units*) identified by spike sorting as sources of spike trains (*SpikeTrains*).

In addition to the recorded data, GNData integrates metadata based on the open metadata Markup Language, odML^14^ (Grewe et al., 2011). odML is an open, flexible and easy to use format to organize metadata in a hierarchical structure of key-value pairs (odML *Properties*). It provides a common *Section* object, which is used to meaningfully group *Properties* according to experimental aspects (Subject, Preparation, Stimulus, Hardware Settings etc.). *Section*s can be nested, enabling a flexible way to organize experimental metadata in a hierarchy that reflects the structure of the experiment. Thus, data annotation can be adapted to the requirements of each specific study. In addition, odML supports standardization by providing common terminologies^15^ —pre-defined odML Section templates for typical experimental aspects to facilitate standardized descriptions of experiments across labs (Grewe et al., 2011).

**Figure 2:**
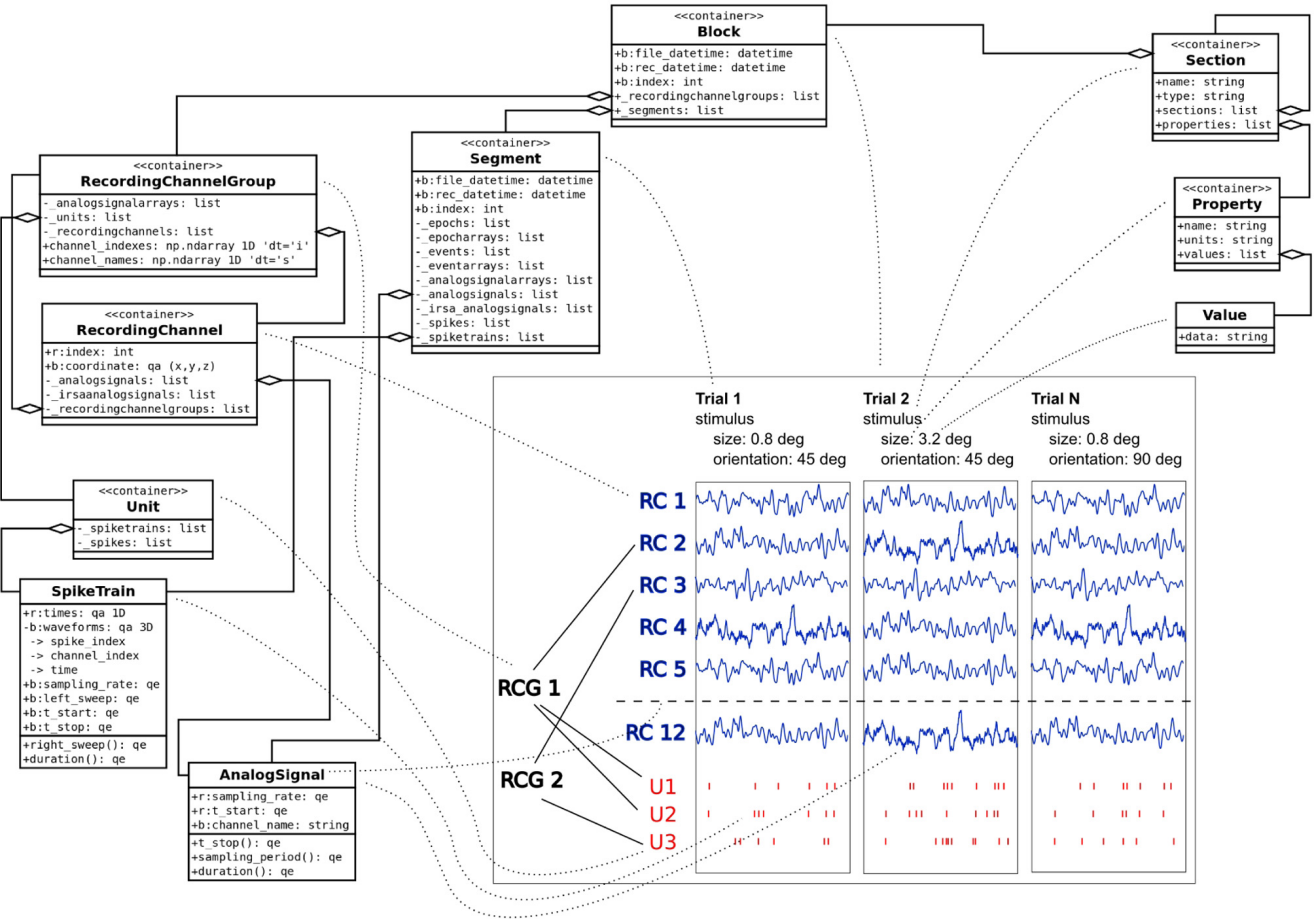
**Example data and metadata structure of an experiment with stimulation changing across trials**. The panel on the bottom right represents the recorded signals in a study that investigates receptive fields of neurons in visual cortex of macaque monkeys. Each trial had its unique stimulus configuration (orientation, size, etc.). Local Field Potentials from different channels (RC1–RC12) were recorded during the experiment; spike trains of single units (U1–U3) were obtained by spike sorting. The dotted lines are used to represent a mapping between experimental entities and their representations in the object model. Neo objects^13^ (left) are used to represent the data part of the experiment. odML^14^ Section, Property and Value objects (top right) describe stimulus metadata, changing from trial to trial within a given experiment.

Combining the Neo and odML concepts in a common object model, GNData integrates data and metadata in a unified framework. An example of the resulting data representation is illustrated in Figure 2.

### 2.2. Common interface to access data and metadata

GNData integrates the Neo data model for electrophysiological data with the flexible odML data annotation under a single API definition. A common API is crucial as it unifies data management approaches, provides a defined way of data access, and makes data and metadata accessible to software tools. Previous approaches (Garcia et al., 2014, The Neuroshare Project^16^) have focused on representation of the recorded data. We complement these designs by integrating the essential methods for data annotation and permissions control, as well as providing a network-accessible implementation.

### 2.3. Client libraries for main computational platforms

The common data API of the GNData platform enables programmatic data access and data management through custom software. To support the use of the data API for everyday data management in the lab, we provide client libraries that communicate with the server via the GNData API, enabling instant data access from the local computational environment. Currently the focus is on Matlab and Python, which are among the most popular computational frameworks in experimental neuroscience. These client libraries^17^ hide the generic API interface from the user and translate the commands and data to representations in the scientist's familiar environment, such as Neo Python objects (Garcia et al., 2014) in the Python client or Matlab structures in the Matlab client (see Appendix in Supplementary Material), thus enabling direct access to the data from the simulation software or analysis script. In addition, a web interface is provided for browser-based access. Enabling different types of data access supports interoperability and makes data access independent of a certain format, language, or platform.

### 2.4. Data sharing

As a multi-user system designed to facilitate collaborative research, GNData provides fine-grained mechanisms for access control and data sharing. Original data is always accessible for its owner. Any subset of data or metadata entities can be shared with selected users, for example collaborators. The ability to instantly access the same data without additional data transfer increases the efficiency of collaborative work. In addition, data can be opened to all users for public access. Thus, it is easy to provide data together with metadata for a data publication, to make selected data available for testing or benchmark purposes, or to release data to the public for re-use.

## 3. Implementation

This section describes the main implementation concepts of the GNData API. A full API reference can be found at the documentation page^18^. A demo environment is available where some of the examples below can be tested to get more detailed overview (Note that object identifiers can be different). Information about the demo environment is provided in the Appendix in Supplementary Material.

### 3.1. REST-FUL interface

The GNData API is built according to the REST principles (Fielding and Taylor, 2002). The REST protocol is designed for data representation supporting caching, scalability and client-server architecture^19^. Many stable open source libraries are available that support REST in different programming languages.

One of the principles of REST is that every object has its permanent location defined via a Uniform Resource Locator (URL): 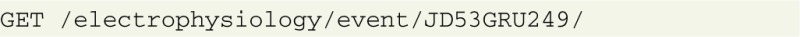 In GNData, URLs defining object locations are designed to have a certain structure. The first part of the URL defines a namespace that corresponds to a particular neuroscientific domain or function. Currently, GNData supports “electrophysiology,” “metadata,” and “datafiles” namespaces, providing electrophysiological data, metadata, and file management functions, respectively. Every namespace includes a set of objects related to this particular namespace. The names of these object types form the second part of the URL. For instance, the “electrophysiology” namespace supports “event,” “spiketrain,” and other object types defined by Neo (see 2.1). The “metadata” namespace supports “section,” “property,” and “value” object types as provided by the odML definition. A unique base-32 (RFC4648) object identifier forms the end part of the object location.

Objects have consistent structured representations (see section 3.2) according to the integrated data model. For all objects, the GNData API supports a number of standard functions like creating, updating and deleting single objects, or making bulk object updates. A standard HTTP GET request selects one or several objects; requests are highly parameterizable, allowing filtering or processing objects in chunks: 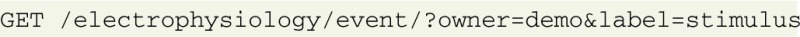 In this example an HTTP GET request queries all event-type objects owned by the user “demo” having a label attribute equal to “stimulus.”

HTTP POST request type with JSON-encoded^20^ data is used for making updates or creating new objects. HTTP DELETE is used to remove objects within the system; removed object will no longer appear in GET responses and will not be available for POST updates. However, removed objects are still accessible after the delete operation as all changes to object status and attributes are being tracked by the system (see section 4.4). Supported operations together with corresponding URL structures are listed in Table 1.

**Table 1:** **Common API actions for every supported HTTP request type (GET, POST, DELETE) and typical request URL structure**.

**URL structure**	**Get**	**Post**	**Delete**
/namespace/object_type/	List objects, apply filters	Create new object or make bulk update	Bulk delete
/namespace/object_type/id/	Access single object	Update single object	Delete single object
/namespace/object_type/id/acl/	Get object permissions	Update object permissions	405 Not Supported

Left column contains structure of the REST URL. Table cells define an action for each URL structure and HTTP request type.

### 3.2. Operations with HTTP request and response

All operations use JSON^20^ as a main request and response format. The JSON format is supported natively by Javascript^21^ and also by many other common programming languages like Python^22^ or Matlab ^23^.

As defined in Table 1, a GET request of the GNData API has the following form: 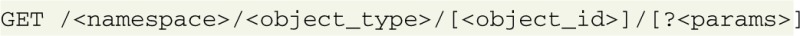 For example, 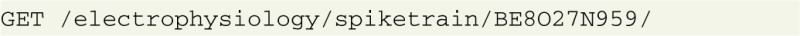 returns the spiketrain object with the identifier “BE8O27N959” in JSON format. In order to create or update objects, a POST request with the same URL syntax is sent. For example, 
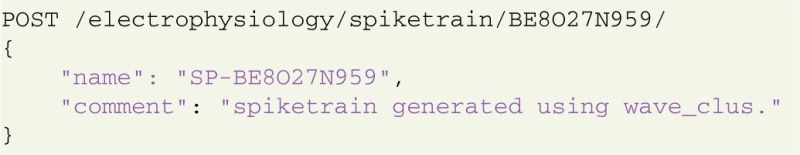
 will make an update fields “name” and “comment” in the spiketrain object with identifier BE8O27N959.

A successful response contains the object represented in JSON format: 
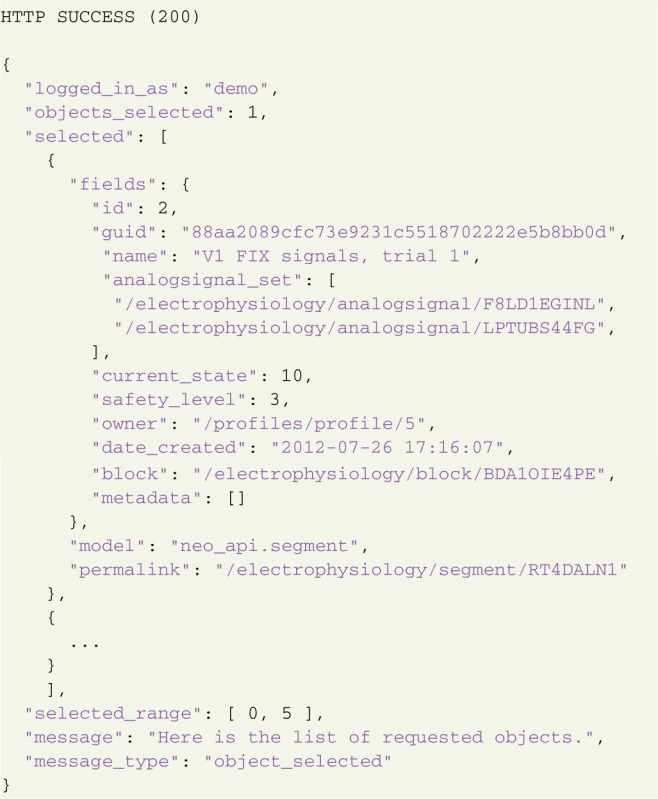
 Exceptions are handled with standard HTTP response codes^24^, such as 404–Object Not Found, 403—Forbidden, 400—Bad Request, 304 - Not Modified, etc., and the response body contains a JSON-formatted message with exception details.

### 3.3. Data handling

GNData uses the HDF5^25^ file format in the backend to store array data. We made several performance tests against popular freely available data storage back-ends (PostgreSQL^26^, MySQL^27^ and HDF5; results not shown) resulting in HDF5 being an optimal solution for managing large data arrays, even with serial file access and fetching of multiple data slices. Every object in the GNData with associated array data (analog signal, spike train, waveform etc.) has the related data stored in HDF5 file. Data can be accessed by downloading a corresponding HDF5 file that contains an HDF5 array in the root of the file.

For data analysis, often only certain selected parts of the recorded data are desired. To reduce data transfer between client and server, a limited data slice can be requested. GNData supports partial data requests for objects with associated data array(s). This works for both single and multiple object requests and is practical when accessing large datasets.

The following request 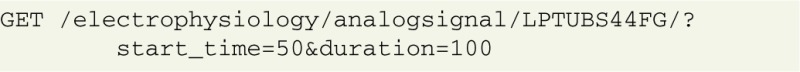 returns data samples falling within the 100 ms time window of the originally recorded signal with ID = LPTUBS44FG starting from 50 ms (units are taken from object attributes). The response (not shown, see example in section 3.2) contains an URL to the corresponding file with a particular slice of array data, 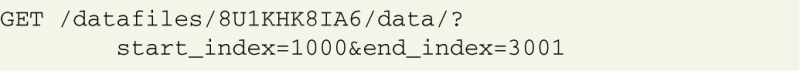 This URL represents a link to a data file containing the data array for the selected analog signal, with parameters indicating the first and the last indexes of this array, needed to create the requested slice. These boundaries are calculated automatically based on the object attributes and their units (start time, sampling rate etc.) and request input parameters (start time, end time, duration etc.). Sending a GET request to this URL will download an HDF5 file containing raw data from the 100 ms time window only. Note that a datafile with array data is no longer dependent on the analog signal and will not contain units or other information, only data itself. All related meta information should be taken from the corresponding object.

### 3.4. Caching

For efficiency, the GNData API is using standard HTTP mechanisms for data caching like e-Tags^28^ and “last-modified” attributes in request headers. Every single change to an object results in a new e-Tag assigned to this object. By default, an object is not served for download if no changes were made and e-Tags of the previously downloaded object and the object on the server match. In this case, a standard 304 HTTP response (“Not Modified”) is returned instead.

### 3.5. Open software and modular structure

**Figure 3:**
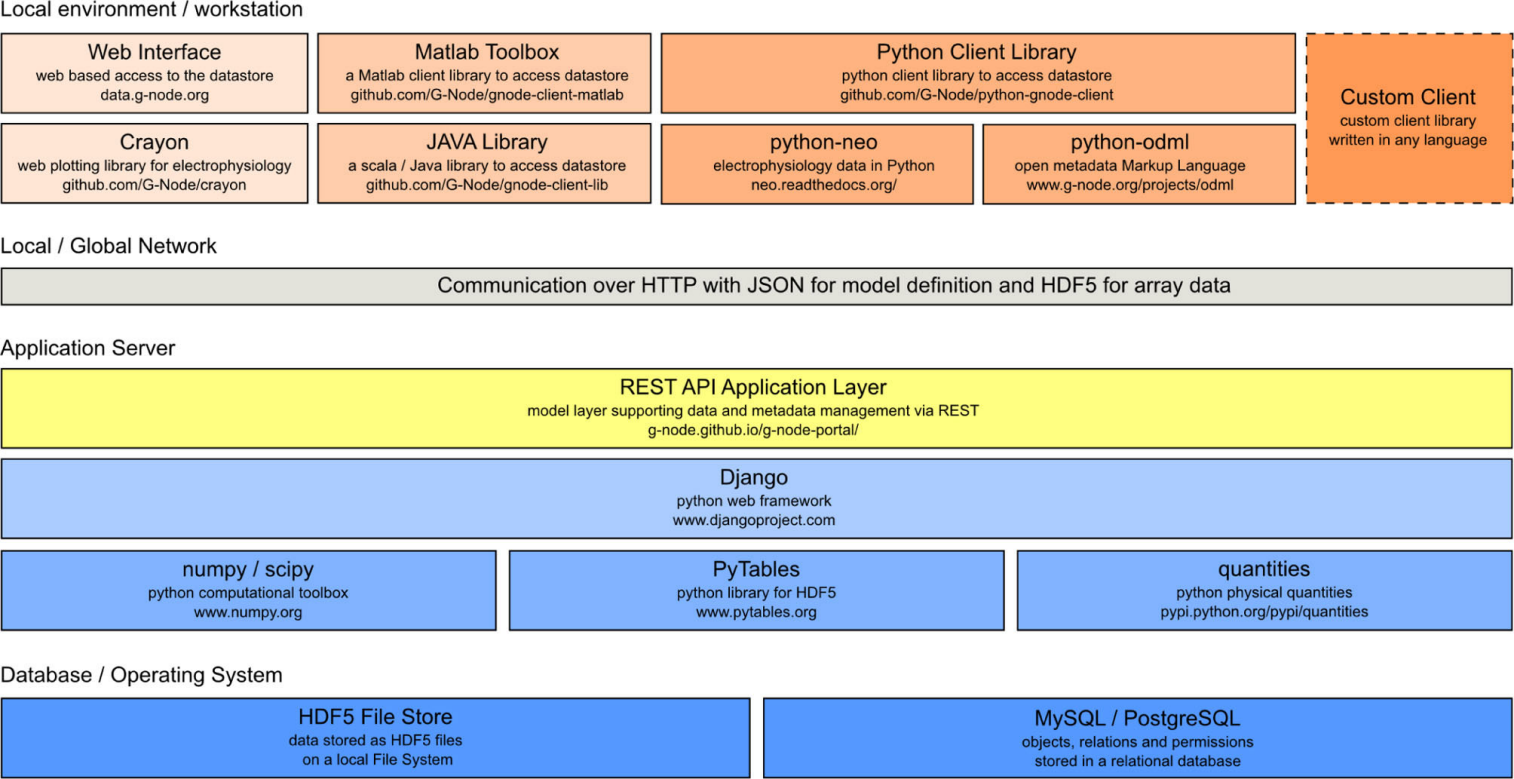
**GNData architecture diagram**. From the bottom: integration of low-level data storage components (dark blue), application server components (light blue) with the REST API (yellow) as the common access interface. Top: Clients such as web interface components, Matlab client components, python client library, self-written custom clients.

GNData is developed as an open source software based on the Django^29^ framework. The framework is designed to be used with relational databases. Concurrent user access, as well as atomicity, consistency and isolation^30^ are implemented on the database level. The software architecture follows a modular principle, so that implementation of new data models into the platform is straightforward^31^. The principal software components are illustrated in Figure 3.

The key programming language used is Python. Python has a growing community in Neuroscience with a large amount of open software available. The GNData project welcomes developers to contribute to the software^32^.

## 4. Key GNDATA features

In this section we describe key features and functional scope of the GNData platform.

### 4.1. Data access with filters

The GNData API provides query mechanism with different filters based on object attributes and relationships. It allows to query a subset of all available objects of a particular type based on certain criteria (equal, greater than, etc.), applied to object attributes. To avoid definition of a new query language, this query mechanism is built on top of the Django querying routine and uses similar concepts and namings^33^. Query parameters, specified in the request URL are directly converted into the request on the Django application level, with addition of certain authorization filters. The following examples illustrate the usage of filters.

**Figure 4:**
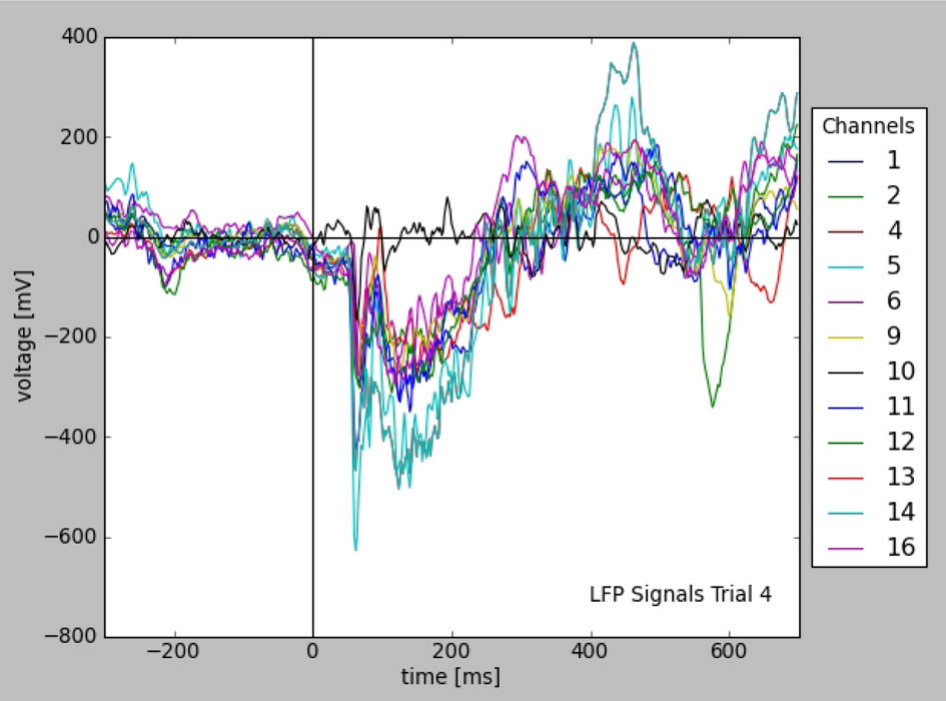
**Plot of LFP responses from a trial selected using certain time and stimulus conditions (see text)**. Note that all informations used for axes, labels, and legend were taken from the stored data and metadata directly.

This HTTP GET request will select metadata properties having “luminance” in their “name” attribute: 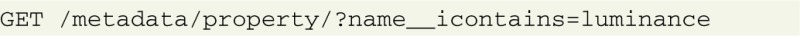 The resulting response contains objects with their identifiers that can be used in another request for related objects (here “value” objects are actual values of related metadata properties): 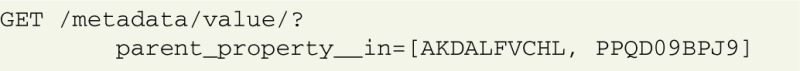 Query conditions on related objects can be directly included in the request parameters. The next example request selects all metadata values of (related) properties with “luminance” in their “name” attribute: 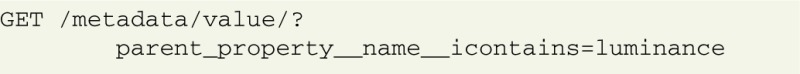 Filters allow to query for a certain subset of the experimental data, which can be used in analysis or visualization. Figure 4 shows the plot of all LFP traces from a certain experimental trial, selected using filters with certain time and stimulus conditions. The query is explained in Appendix (Supplementary Material) in more detail.

A full query reference is available at the project documentation page.

### 4.2. Unified organization of data and metadata

GNData provides a common set of objects representing electrophysiological (experimental and/or simulated) data, together with an object model for flexible metadata description. The GNData API allows to establish meaningful connections between data and metadata objects. In particular, data objects can be hierarchically grouped (using odML *Sections*) to achieve an efficient organization. Any data object can be annotated by linking with the appropriate metadata objects, thus achieving a comprehensive data annotation for data selection and reproducibility. For example, consider an experiment where stimulus parameters change from trial to trial. In that case, for every experimental trial the appropriate stimulus property has to be indicated, which can be achieved by annotating each Neo *Segment* representing a trial to the appropriate metadata values.

Annotation is done by sending an HTTP POST request with the references to the metadata values and the target object for annotation: 
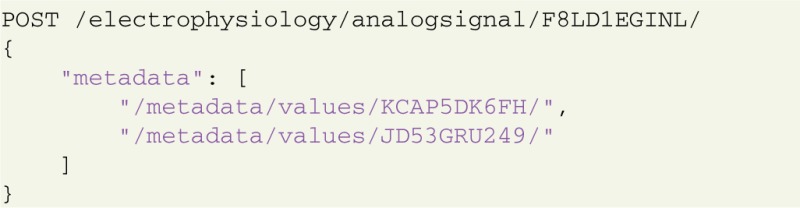
 In this example, an analog signal object with ID = F8LD1EGINL is annotated with certain metadata values (KCAP5DK6FH and JD53GRU249). Required values and their IDs can be pre-selected with another request using appropriate conditions and parameters (see section 4.1). These connections enable the researcher not only to identify the experimental context for a given data structure, but conversely also to query data by specific metadata. The following request selects all analog signal objects that have the above defined values as their metadata: 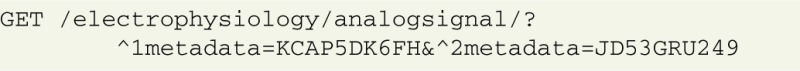 In general, this allows using annotated metadata in requests for data object of any type.

### 4.3. Data sharing and collaboration

The GNData system provides a multi-user environment that facilitates collaborations where researchers need common access to datasets. Control of data access permissions is achieved using Access Control Lists (ACL), which provides several access levels for each object. By default, every object created in the system is private and only accessible for its owner. The owner of an object can make it accessible for an individual or group of collaborators, or open it for access to all users. Thus, data sharing can be done with a simple command, avoiding any data duplication or transfer.

GNData supports both read-only and read-write permissions for individual shares. The whole study can be opened for experimentalists for contribution with experimental recordings, while certain experimental trials can be made read-only for collaborators who perform data analysis.

Each object's current ACL is available for the object owner at a specific URL: 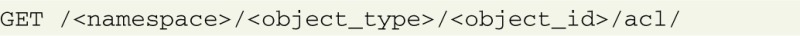 The structure of the ACL in JSON format contains its global sharing level and the list of users having individual access: 
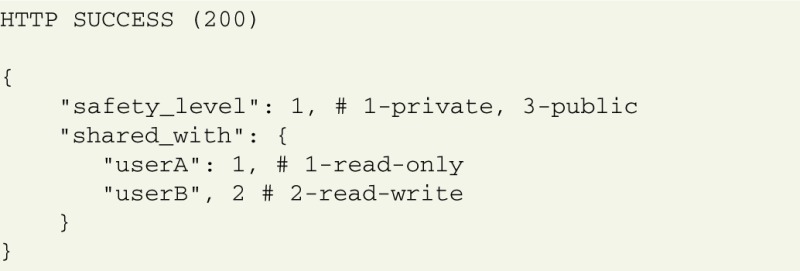
 An authorized POST request to this URL with the request body containing new ACL configuration updates the object permissions.

### 4.4. Versioning

To support reproducibility, GNData implements object versioning mechanisms where all changes to any object are saved, and a user can always go back in time to the corresponding version of the data.

Requesting a certain version of an object is done by adding the “at_time” parameter to the GET request. The following example requests an object as it was at September, 15th 15:36:55: 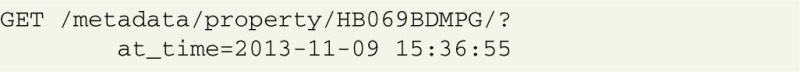


## 5. Discussion

We presented GNData, a data management system with an open API for electrophysiological data. GNData unifies organization of data and corresponding metadata and provides data access for researchers within a lab as well as for collaborators, directly from their computation environments. Efficient organization of data and metadata saves time for data access and facilitates data exchange and collaboration. Moreover, programmatic data access enables automatization of many steps in data collection and data organization, thus facilitating data analysis and collaborative research.

Key principle of the GNData architecture is an API that separates the user application from the storage backend and represents a consistent interface for accessing electrophysiological data. A common interface saves development and maintenance efforts and creates interoperability, faciliating application of tools and integration of software solutions. The GNData API combines a common representation for recorded data and a flexible metadata schema that is suitable to annotate data from any kind of experiment. This concept is independent of the REST implementation. Implementations in other programming languages and on different technologies are easily possible.

The GNData API includes basic functions for querying data using different filters applied to object attributes. More extensive search capabilities and support for complex queries would be desirable for data retrieval. Extended functionality based on existing open-source solutions (e.g., Lucene^34^, Xapian^35^, Minion^36^, Elasticsearch^37^ etc.) will be included in future releases.

The GNData platform provides a standardized data representation, but original recorded data can come from files in various formats. Using the G-Node Python client (Sobolev et al., 2014, see also Appendix in Supplementary Material) users can utilize the Neo I/O modules (Garcia et al., 2014) to read the data into Neo objects before uploading. However, ideally the central data server would provide this data conversion, so that users can upload their data in files which are automatically extracted to corresponding data structures on the platform. For this purpose, integration of the Neo I/O libraries into the GNData is under development.

Currently GNData is focused on electrophysiological data. Scalability of the data model and the Client-Server approach, however, allow straightforward extension to account for data from other fields of neuroscience. Neuromorphological data, imaging data or other types of data could be integrated simply by specifying the appropriate data models. The INCF Task Forces on Electrophysiology^38^ and Neuroimaging^39^ are currently working on standard data models and formats for the respective types of data (Teeters et al., 2013). Those standards will be integrated in the GNData platform as they are released. Likewise, to support the entire data processing workflow in the laboratory, results from data analysis need to be accommodated as well. This kind of extension will be introduced as one of the next steps.

GNData is developed as an open source project available at the public G-Node Github account^40^. The project is open to contribution from neuroscientists or members from other scientific fields.

## Funding

Supported by the Federal Ministry of Education and Research (Grants 01GQ0801 and 01GQ1302).

### Conflict of interest statement

The authors declare that the research was conducted in the absence of any commercial or financial relationships that could be construed as a potential conflict of interest.

## Footnotes

^1^http://neurodatabase.org

^2^https://senselab.med.yale.edu

^3^http://www.neuinfo.org

^4^http://incf.org

^5^http://crcns.org

^6^http://www.carmen.org.uk/

^7^http://www.carmen.org.uk/standards

^8^https://portal.g-node.org/data/

^9^http://incf.org/dataspace

^10^http://www.frontiersin.org/10.3389/conf.fncom.2012.55.00033/event_abstract

^11^https://www.humanbrainproject.eu/

^12^http://www.nih.gov/science/brain/

^13^http://neuralensemble.org/neo/

^14^http://www.g-node.org/projects/odml

^15^http://www.g-node.org/projects/odml/terminologies

^16^http://neuroshare.sourceforge.net/API-Documentation/NeuroshareAPI-1-3.htm

^17^http://g-node.github.io/python-gnode-client, http://github.com/G-Node/gnode-client-matlab

^18^http://g-node.github.io/g-node-portal/

^19^http://en.wikipedia.org/wiki/Client-server_model

^20^http://www.json.org/

^21^http://en.wikipedia.org/wiki/JavaScript

^22^http://docs.python.org/2/library/json.html

^23^http://www.mathworks.com/matlabcentral/fileexchange/20565-json-parser

^24^http://www.w3.org/Protocols/HTTP/HTRESP.html

^25^http://www.hdfgroup.org/HDF5/

^26^http://www.postgresql.org/

^27^http://www.mysql.com/

^28^http://en.wikipedia.org/wiki/HTTP_ETag

^29^https://www.djangoproject.com/

^30^http://en.wikipedia.org/wiki/ACID

^31^https://docs.djangoproject.com/en/dev/topics/db/models/

^32^https://github.com/G-Node/g-node-portal/

^33^https://docs.djangoproject.com/en/dev/topics/db/queries/

^34^http://lucene.apache.org/

^35^http://xapian.org/

^36^https://minion.java.net/

^37^http://www.elasticsearch.org/

^38^http://www.incf.org/programs/datasharing/electrophysiology-task-force

^39^http://www.incf.org/programs/datasharing/neuroimaging-task-force

^40^https://github.com/G-node
