# Supplementary material for: Integrated platform and API for electrophysiological data
Source: Front Neuroinform. 2014 Apr 23;8:32. doi: 10.3389/fninf.2014.00032 (PMC4005939; doi:10.3389/fninf.2014.00032)
Supplement: Supplementary file 1 [file Presentation1.PDF]

APPENDIX: EXAMPLES OF DATA ACCESS USING WEB BROWSER, MATLAB AND PYTHON

In this Appendix we provide several use case examples of the GNData platform with application to real experimental data. For practial illustration we use G-Node client libraries for Matlab and Python to show data management operations directly within the computational framework. Additionally, we provide a short description on web browser access for overview purposes. For proper operation a server part should be available. A demo environment is provided by the G-Node at `http://test.g-node.org`, which can be accessed for testing or introductory purposes without user account registration.

In the following examples, we consider a typical experimental study in which responses from neurons in the visual cortex of macaque monkeys are recorded (Teichert et al., 2007). These examples assume a dataset containing recorded neural responses that is pre-loaded on the demo server.

1 ACCESSING REST INTERFACE WITH WEB BROWSER

For this demo we recommend a combination of a web browser with a REST client as a framework to execute HTTP requests (for example, Google Chrome[1] with Postman[2]). Alternatively, HTTP requests can be also executed directly from the web browser's address bar but with the loss of human-readable response formatting.

Authentication for the demo user is done with the following POST request:

```
POST /account/authenticate/
{
    "username": "demo",
    "password": "demo"
}
```

The browser saves authentication and session information automatically, allowing to execute further API requests.

As a short example, the following HTTP request selects all experimental trials created on November, 9th that have 'saccade' in their name.

```
GET /electrophysiology/segment/?date_created__month=11&date_created__day=9&name__icontains=saccade
```

Further requests can be executed according to the GNData API documentation[3] or the examples presented in this manuscript.

[1] http://google.com/chrome
[2] http://chrome.google.com/webstore/detail/postman-rest-client/fdmmgilgnpjigdojojpjoooidkmcomcm
[3] http://g-node.github.io/g-node-portal/

2 ACCESSING DATA WITH MATLAB

The Matlab library is a free package accessible at the G-Node Github repository[4]. Installation instructions and package documentation are available at the project wiki page[5].

To start using the library it is necessary to import the ”gnode” functions into the current scope and initialize a session:

```
% move all library functions into scope
import gnode.*;

% initialize session as user "demo" with password "demo"
g = init('demo', 'demo', 'test.g-node.org');
```

Data at GNData is stored in a set of object types tailored to electrophysiology, such as blocks, segments, recording channels, signals etc. In the Matlab environment, these objects are represented as structures. Here is a basic example for creating and uploading such an object.

```
% create a structure containing signal data
signal = make_dummy(g, 'analogsignal');

signal.name = 'New signal';
signal.sampling_rate = struct('units', 'Hz', 'data', 12000);
signal.t_start = struct('units', 'ms', 'data', 0);
signal.signal = struct('units', 'mV', 'data', [3.45, 1.34, 1.45, 4.22]);

% creates object on the server and returns its unique ID
new_object = create(g, signal);
```

Object retrieval is equally straightforward and supports various access methods (e.g. by object type, by array of IDs, using filters or a numeric range). Here is an example that requests all time segments representing experimental trials that were created on November, 9th and have ’saccade’ in their name:

```
% query LFP data using filters
filter1 = {'date_created__month', '11'};
filter2 = {'date_created__day', '9'};
filter3 = {'name__icontains', 'saccade'};

segments = browse_search(g, 'segment', filter1{:}, filter2{:}, filter3{:});
```

The following example downloads one of the time segments with all related analog signal and spike time data:

```
segment_with_data = get_cascade(g, segments{1})
```

More advanced operations (e.g., updates, download queues, batch object creation and upload, and so on) are described in the reference.

[4] https://github.com/G-Node/gnode-client-matlab/

[5] https://github.com/G-Node/gnode-client-matlab/wiki

3 ACCESSING DATA WITH PYTHON

The Python Client library (Sobolev et al., 2014) is a free package accessible at the G-Node Github repository[6] as well as at the standard python package repository[7]. Installation instructions are contained in the attached documentation as well as online at the project documentation page[8].

To start using the library it is necessary to import the session class and initialize a session:

```
# import core session class and model descriptions
from gnodeclient import session, Model

# Initialize session as user "demo" with password "demo"
g = session.create(username="demo", password="demo", location="http://test.g-node.org")
```

A session object provides the main interface to work with data and metadata. Like with the Matlab library example above, we demonstrate how to select a certain experimental trial using several criteria. In particular, the following script requests all time segments representing experimental trials that were created on November, 9th and have 'saccade' in their name.

```
filters = {
    'date_created__month': 11,
    'date_created__day': 9,
    'name__icontains': 'saccade'
}

time_segments = g.select(Model.SEGMENT, filters)
```

This results in a number of experimental trials, saved on that day. The Neo data model (Garcia et al., 2014) assumes that time segments contain analog signal or event type data. For illustration, the script below plots all LFP traces from the first trial of the selection.

```
# take a first segment from the selection
lfp_data = time_segments[0].analogsignals

# plot LFP data from a certain trial (downloaded data is cached)
from matplotlib import pylab as pl

lfp = pl.subplot(111)
for s in lfp_data:
    lfp.plot(s.times, s, label=s.recordingchannel.index)

s1 = lfp_data[0] # one of the signals
pl.xlabel("time [%s]" % s1.times.units.dimensionality.string) # set X units
pl.ylabel("voltage [%s]" % s1.units.dimensionality.string) # set Y units

# [...] # commands for axes and legend omitted

pl.show()
```

[6] https://github.com/G-Node/python-gnode-client

[7] https://pypi.python.org/pypi

[8] https://g-node.github.io/python-gnode-client/

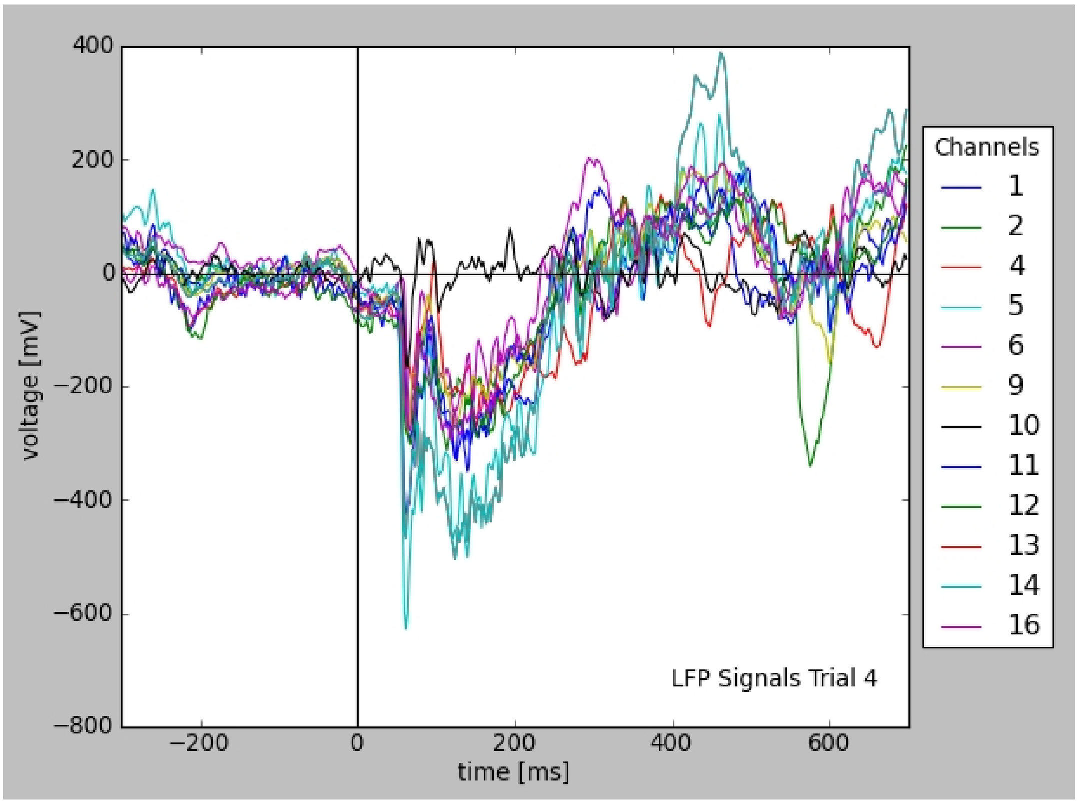

Figure 1. Plot of LFP responses from a trial selected using certain time and stimulus conditions (see text). Note that all informations used for axes, labels, and legend were taken from the stored data and metadata directly.

Further examples as well as the full library reference are available at the project documentation page.

REFERENCES

Teichert, T., Wachtler, T., Michler, F., Gail, A., and Eckhorn, R. (2007) Scale-invariance of receptive field properties in primary visual cortex. *BMC Neuroscience* 8 :38. doi:doi: 10.1186/1471-2202-8-38.

Sobolev, A., Stoewer, A., Pereira, M., Kellner, C. J., Garbers, C., Rautenberg, P. L., et al. (2014) Data management routines for reproducible research using the G-Node Python Client library. *Front Neuroinform* 8.

Garcia, S., Guarino, D., Jaillet, F., Jennings, T. R., Pröpper, R., Rautenberg, P. L., et al. (2014) Neo: an object model for handling electrophysiology data in multiple formats. *Frontiers in Neuroinformatics* 8. doi:10.3389/fninf.2014.00010.
